# A Reward

**Published:** 1860-03

**Authors:** 


### A REWARD.

FIFTEEN cents will be paid to the finder and deliverer at Forty-Two Irving Place, New-York, of a roll containing, first, a piece of a newspaper covering a copy of the February Number of HALL'S JOURNAL OF HEALTH, and eleven smaller rolls of the off-sheets of old letters, etc., containing a great variety of hieroglyphics pertaining more or less directly to the following subjects:

| | |
|---|---|
| Knowing and Unknown. | "Surfeiting and Drunkenness." |
| Northern Butcheries. | Life Saved. |
| Health and Palmerston. | Faulty Construction of Churches. |
| Fever and Ague. | Instinct of Appetite, etc., etc. |

Ten cents will be paid for the JOURNAL alone, or five cents and no thanks for the manuscript.

RAILROAD SAFETY.—Of the millions of passengers traveling in the cars of the New-Jersey Railroad, in a quarter of a century, not a single life has been lost of any passenger who was in his proper place at the time of any accident. The Camden and Amboy road has never been operated with greater regularity, safety, and comfort to passengers than now.

SEWING-MACHINES.—The wife of one of the most prominent and influential citizens said yesterday, if Wheeler and Wilson's Sewing-Machines had the patent needle threader, requiring little use of the eye, it needed nothing more to be a perfection. Why don't they have it?

DR. JOHN ELLIS has published, at room No. 20 Cooper Institute, New-York, a dollar book of 348 pages on *The Avoidable Causes of Disease, Insanity, and Deformity*, under the motto: "The prevention of disease is better than its cure." It contains a vast amount of interesting, reliable, and practical information. We recommend it because he is worse than a homœopath: he gives no medicine at all. H. B. Price, publisher, 884 Broadway, keeps it. Postage ten cents extra.

